# Invasive Breast Cancer of No Special Type With Osteoclast-Like Giant Cells: A Cytological Clue Providing the Final Diagnosis for Histology

**DOI:** 10.7759/cureus.58518

**Published:** 2024-04-18

**Authors:** Tristan Veekmans, Birgit Weynand, Giuseppe Floris

**Affiliations:** 1 Department of Pathology, KU Leuven-University of Leuven, University Hospitals Leuven, Leuven, BEL

**Keywords:** lymph node cytology, multinucleated cells, cytological-histological correlation, osteoclast-like giant cells, breast cancer

## Abstract

Breast cancer associated with osteoclast-like giant cells (OGCs) refers to a morphological pattern of invasive breast carcinoma of non-special type. Their presence is sometimes subtle, but OGCs can be appreciated both histologically and immunohistochemically. The origin of OGCs as well as their implication for prognosis remain debated. We describe the case of a 65-year-old woman, wherein the presence of OGCs in the fine-needle aspiration cytology of a metastatic axillary lymph node suggested the final diagnosis on histology. The differential diagnosis is broad, and here we provide evidence for strict cytological-histological correlation when dealing with unusual breast lesions.

## Introduction

Invasive breast cancer with the presence of osteoclast-like giant cells (OGCs) is observed in fewer than 2% of total breast cancer cases [1]. OGCs have been described in all types of breast cancer such as tubular carcinoma, metaplastic carcinoma, and even ductal carcinoma in situ (DCIS), but most commonly in cribriform carcinoma [2,3]. OGCs are frequently associated with a highly cellular stroma with various degrees of extravasated erythrocytes which may mask their presence. The invasive component generally shows a high expression of hormone receptors and a low-to-moderate grade of differentiation. Their significance for prognosis is unclear [2]. Moreover, their origin remains a subject of debate [4-8].

The diagnosis, especially on cytology, can be challenging as the presence of multinucleated giant cells (MGCs) in the breast can be rather subtle, aside from the fact that it can occur in both malignant and benign conditions [1,9].

We present a case in which the final histological diagnosis of invasive breast cancer of no special type (IBC-NST) associated with OGCs was suggested by the findings obtained from the fine-needle aspiration cytology (FNAC) of the axillary lymph node.

## Case presentation

**Figure 1:**
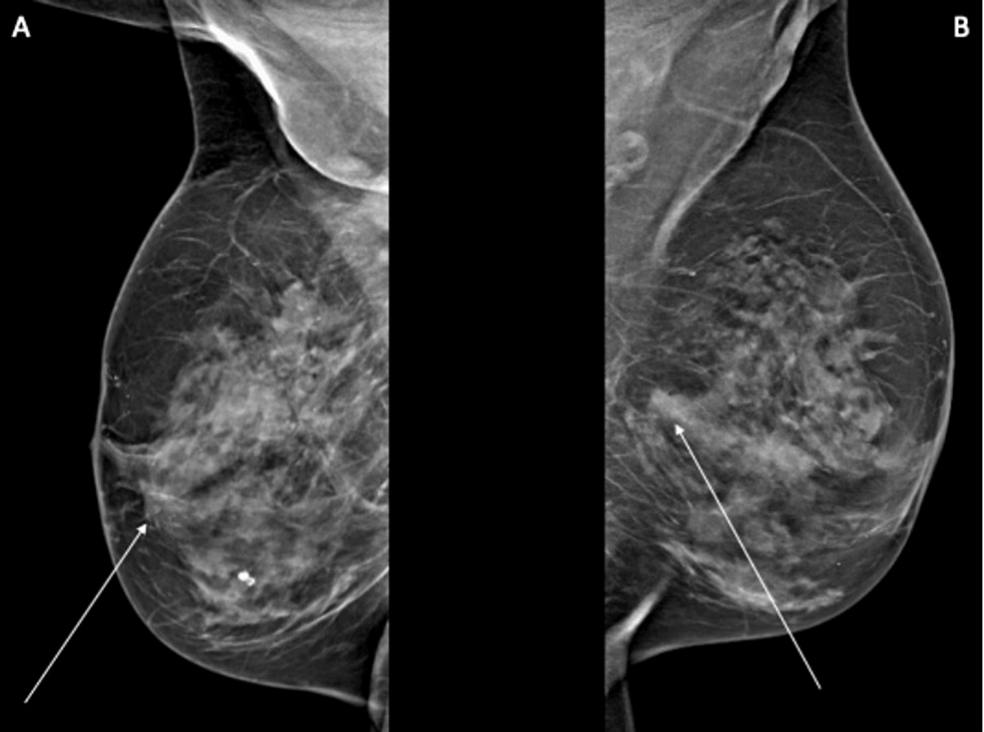
X-rays of both breasts. (A) Right breast: The image shows a periareolar lesion with ill-defined borders measuring approximately 24 × 24 × 24 mm (arrow). (B) Left breast: The image shows a lesion located deep in the breast, prepectoral, with ill-defined borders measuring approximately 10.6 × 5.4 × 6.8 mm (arrow).

**Figure 2:**
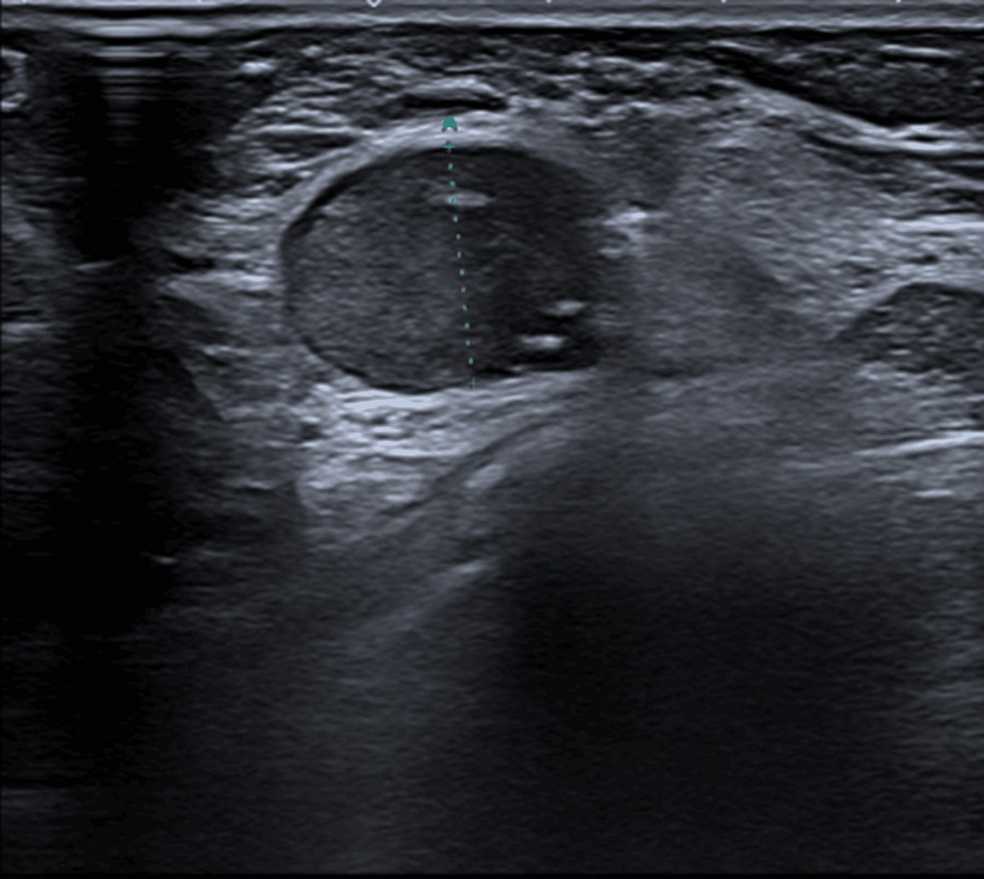
Ultrasound of the left axilla. The image shows one of the suspicious lymph nodes in the left axilla. Fine-needle aspiration cytology of one of these lymph nodes was performed.

A 65-year-old Middle Eastern woman presented to her general practitioner with complaints about episodic pain in her right arm for several weeks. The patient reported no significant medical history, including other systemic illnesses such as diabetes. Upon further clinical investigation, the patient reported no other symptoms or complaints. Clinical examination showed palpable masses in the right and left breast as well as enlarged axillary lymph nodes in the left axilla, prompting further investigation by ultrasound and mammography. Radiology showed bilateral suspicious lesions (Figure 1) associated with bilaterally enlarged lymph nodes in the axilla (Figure 2). A core needle biopsy (CNB) of both breast lesions was taken, as well as an FNAC of one of the enlarged lymph nodes in the left axilla.

**Figure 3:**
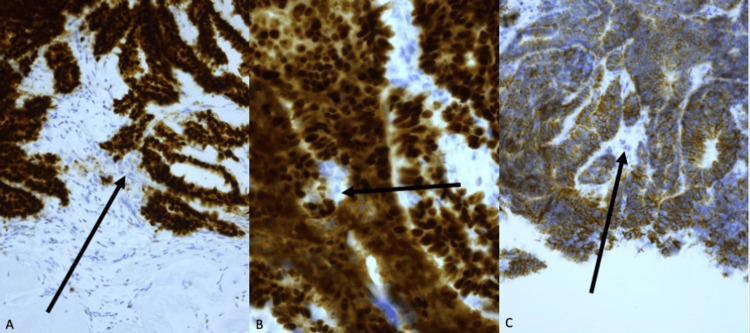
Estrogen receptor, progesterone receptor, and HER2 immunohistochemistry. (A) Estrogen receptor showed strong immunoreactivity in the tumor (Allred score 8) and negative staining in the osteoclast-like giant cells (arrow). (B) Progesterone receptor showed strong immunoreactivity in the tumor (Allred score 8) and negative staining in the osteoclast-like giant cells (arrow). (C) HER2 was scored as 2+. HER2 was negative in the osteoclast-like giant cells (arrow).

**Figure 4:**
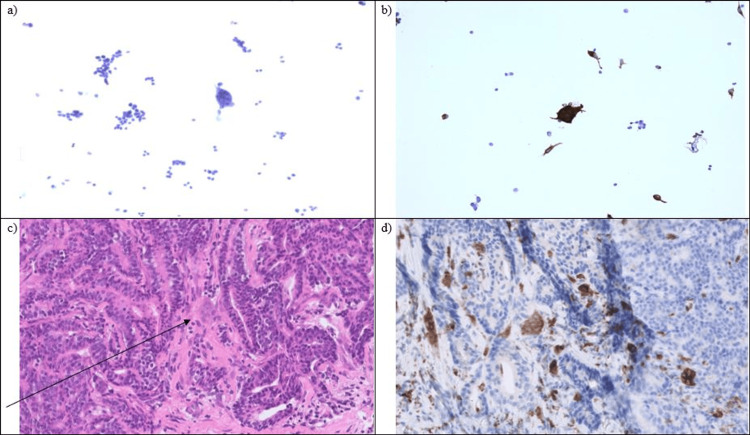
Cytology and histology findings. (a) Presence of multinucleated cells in the cytology specimen. (b) Multinucleated cells showing immunoreactivity for CD68. (c) Core needle biopsy with invasive breast carcinoma of no special type and the presence of multinucleated giant cells (arrow). (d) Multinucleated cells showing immunoreactivity for CD68.

Under the microscope, at first sight, both breast lesions revealed the presence of a well-to-moderately IBC-NST associated with moderately differentiated DCIS. The presence of DCIS strongly pointed toward a bilateral primary breast carcinoma. Both tumors showed strong nuclear expression of estrogen receptor (ER, Allred quick score 8/8), progesterone receptor (PR, Allred quick score 8/8), and equivocal HER2 expression (score 2+) with subsequent negative fluorescence in situ hybridization for HER2 gene amplification (Figure 3). The FNAC from the enlarged lymph node in the left axilla showed a metastatic deposit of low-grade carcinoma in association with numerous multinucleated OGCs. This finding prompted us to revise the histology of CNB, which revealed in the CNB of the left side the subtle presence of OGCs that were initially overlooked. Further immunohistochemistry confirmed the presence of OGCs in the FNAC and CNB of the left side (Figure 4). CD68 and cytokeratin stains were performed on both the CNB of the breast and the FNAC of the axillary lymph node. Cytokeratin was strongly positive only in the tumor cells and negative in the giant cells, while CD68 was strongly positive only in the giant cells and negative in the tumor cells. No OGCs were present on the right side after careful revision. Eventually, the diagnosis of IBC-NST with associated OGCs was proposed, excluding other etiologies in the axilla. Further treatment of the patient was performed in another institution.

## Discussion

MGCs can be frequently encountered in the diagnostic workup of breast lesions and their related regional lymph nodes. Their presence may be seen in benign as well as malignant lesions, prompting the investigation of the underlying etiology when MGCs are encountered [2,3,5]. Here, the presence of MGCs with OGC features in the axilla was easily explained upon a careful review of the CNB of the homolateral breast lesion which showed the presence of IBC-NST with OGCs.

While in the past editions of the World Health Organization (WHO) classification of tumors of the breast, carcinomas with OGCs were regarded as a specific variant of breast carcinoma, in the current WHO Blue Book, the presence of OGCs is simply considered a special morphological pattern of IBC-NST [10]. Indeed, OGCs have been described in all histological subtypes of invasive breast carcinoma and even DCIS [2,3,5], but they are most frequently associated with low-grade tumors, showing high expression of hormone receptors (also referred to as luminal A-like breast tumors).

Alongside the presence of OGCs, these tumors are often associated with an inflammatory and hypervascular stroma which cannot be explained due to biopsy changes [2,4]. Apart from malignant and benign disease in the breast [1,9], OGCs have also been described in cancers in other organs such as the thyroid, liver, and pancreas [3,11].

Apart from the histological appearance, OGCs can be appreciated immunohistochemically [12]. OGCs typically stain for CD68, as well as for NSE, lysozyme, acid phosphatase, and CD163, while they are typically negative for ER, PR, HER2, S100, actin, E-cadherin, and alkaline phosphatase [9,12].

The underpinning mechanisms involved in the development of OGCs in breast carcinomas remain a subject of debate. Some evidence suggests that OGCs are merely a reaction of the surrounding tissue to the malignant tumor cells stimulated by the secretion of cytokines and growth factors. Others have hypothesized that OGCs develop in response to a pro-tumoral micro-environment losing antigen-presenting features. On the contrary, it is well established that OGCs derive from macrophages or stromal histiocytes, and that their presence does not affect the prognosis of the related carcinoma [4,5,7,8].

In the absence of a direct correlation with histology, the presence of OGCs in cytologic samples of breast-related lesions may pose significant challenges. When considering other malignancies in the breast, the differential diagnosis can be difficult, especially in the case of metaplastic breast carcinoma with OGCs or metaplastic breast carcinoma with heterologous mesenchymal differentiation with bone formation [12,13]. In this case, it is important to take into account the morphology of the tumor cells and complement it with a broad spectrum of high and low-molecular-weight cytokeratin to confirm the epithelial origin of the lesion. To qualify for heterologous mesenchymal differentiation, the finding of OGCs alone is not enough, warranting extensive sampling of the surgical specimen [13]. In these cases of metaplastic breast carcinoma with heterologous mesenchymal differentiation, OGCs are considered active components of the tumor and therefore malignant when showing nuclear atypia [13]. Another important finding on cytologic aspirates derived from lymph nodes of breast cancer patients may be the presence of megakaryocytes as a consequence of extramedullary hematopoiesis, which may occur as a consequence of a related hematologic disorder or in the context of treatment with hematopoietic growth factors (e.g., during chemotherapy treatment) [14,15].

Apart from malignancy, there is a broad differential diagnosis of benign entities as well as inflammatory diseases in the breast which can present with MGCs [16]. Granulomatous mastitis (GM) is a chronic, inflammatory disease known to mimic cancer, both clinically and radiologically [17,18]. GM is subdivided in specific versus non-specific or idiopathic GM based on whether the primary cause of the inflammation is known [17,18]. Specific causes for granulomatous infections in the breast and related regional lymph nodes include infectious diseases such as tuberculosis and parasitic and fungal infections such as cryptococcosis, blastomycosis, and filarial infections. These infections are associated with other symptoms as well as acute inflammation and (necrotizing) granulomas in the biopsy or FNAC. Especially tuberculosis is known to present with necrotizing granulomas. Stains for microorganisms such as Ziehl-Nielsen for mycobacteria or fungal stains can help identify the etiological organism [17]. Wegener granulomatosis, giant cell arteritis, diabetes mellitus, sarcoidosis, as well as foreign body reactions to, for instance, silicone, can present with multinucleated cells in the breast. For all of these causes of specific GM, correlation with the clinical presentation as well as the history of the patient is key [19].

Non-specific or idiopathic GM is a granulomatous inflammation in the breast for which there is no identifiable cause, but should always remain a diagnosis of exclusion [17,18].

## Conclusions

The finding of MGCs in a lymph node FNAC provides a broad differential diagnosis. Despite several benign entities, the finding of OGCs in an axillary lymph node should trigger additional attention in order not to miss an underlying malignancy. Correlation with clinical and radiological findings is paramount.
